# Early Needs Assessment in Patients With Non‐Specific Symptoms of Cancer: An Explorative Study

**DOI:** 10.1002/pri.70136

**Published:** 2025-11-29

**Authors:** Helene Nørgaard Kristensen, Rikke Aarhus, Jannie Rhod Bloch‐Nielsen, Thomas Maribo, Anne Mette Schmidt

**Affiliations:** ^1^ Medical Diagnostic Center University Clinic for Innovative Patient Pathways, Silkeborg Regional Hospital Silkeborg Denmark; ^2^ Medical Diagnostic Center, Department of Physiotherapy and Occupational Therapy Silkeborg Regional Hospital Silkeborg Denmark; ^3^ VIVE, The Danish Center for Social Science Research Åbyhøj Denmark; ^4^ DEFACTUM Aarhus Denmark; ^5^ Department of Public Health Aarhus University Aarhus Denmark

**Keywords:** cancer, disability and health, international classification of functioning, needs assessment, rehabilitation

## Abstract

**Background and Purpose:**

The incidence of cancer is growing causing considerable morbidity and mortality. Despite knowledge about the level of disability in patients with cancer, recognition of the value of rehabilitation and recommendations as well as adoption of systematic needs assessments is lacking. The aim was to describe patients with non‐specific symptoms of cancer following the adoption of systematic needs assessment among those diagnosed with cancer and to evaluate the perspectives of patients, physiotherapists and other health professionals on needs assessment.

**Methods:**

An explorative study including patients undergoing cancer diagnostics and health professionals in a cancer diagnostic clinic was conducted. Needs assessments among 39 patients with cancer, five individual patient interviews and a focus group interview with five health professionals were conducted between October 2021 and October 2022. Interviews were analysed using inductive thematic analysis.

**Results:**

In total, 39 of 55 patients completed needs assessment, and among these, 27 patients reported having various needs related to disability. Overall, patients and health professionals found early rehabilitation meaningful and the specific needs assessment form was also useful to provide information and guide conversations. Patients and health professionals also addressed barriers and suggestions for optimising efforts in future practice in relation to needs assessment for patients diagnosed with cancer.

**Discussion:**

We characterised 55 patients diagnosed with cancer and identified their rehabilitation needs. Further, we documented that early systematic needs assessment conducted by physiotherapists and occupational therapists proved valuable and was generally well‐received by both patients and health professionals. It underscores the significance of early rehabilitation with emphasis on daily life and identifies key areas where improvements can be made to better support rehabilitation of patients diagnosed with cancer.

## 1 Introduction

Worldwide, the incidence of cancer is growing causing considerable morbidity and mortality (Sung et al. 2021). Cancer diagnostics and treatment have thus been conducted in accelerated organ‐specific pathways implemented in Australia and several European countries since the early 2000s (Jensen et al. 2022). About half of the patients with cancer present with vague or non‐specific symptoms (Næser et al. 2017) such as weight loss, fatigue, pain, and loss of appetite (Jensen et al. 2022). In Denmark and Sweden, diagnostic pathways have thus been implemented for patients with non‐specific cancer symptoms (Sundhedsstyrelsen 2022; Stenman et al. 2019). The prevalence of cancer among patients referred to these pathways ranges from 11% to 35% (Jensen et al. 2022).

More than 60% of patients living with cancer (Hansen et al. 2013; Holm et al. 2012; Jørgensen et al. 2017; Neo et al. 2017; Thorsen et al. 2011) experience physical, psychological, social and spiritual/religious disability or a combination of those (Sundhedsstyrelsen. Forløbsprogram for rehabilitering og, 2018). The challenges caused by disability depend on cancer type and prognosis as well as patient‐related factors such as age, comorbidity, and lifestyle (Hansen et al. 2013; Sundhedsstyrelsen. Forløbsprogram for rehabilitering og, 2018). Rehabilitation efforts address disability‐related challenges (Sundhedsstyrelsen. Forløbsprogram for rehabilitering og, 2018; Cieza et al. 2021), and clinical guidelines in cancer include recommendations for rehabilitation (Stout et al. 2021). Despite the widespread recognition of the important role of rehabilitation to address disability‐related challenges, previous studies have reported unmet patient needs and low referral to rehabilitation (Holm et al. 2012; Sundhedsstyrelsen. Forløbsprogram for rehabilitering og, 2018; Stout et al. 2021; Silver 2017; Rosario‐Concepción et al. 2021). Expertise within disability and rehabilitation typically falls within the physiotherapy and occupational therapy professions, making them crucial stakeholders in needs assessment (Rijpkema et al. 2020).

In accordance with international recommendations (Alfano et al. 2016), Denmark has introduced a disease management program for rehabilitation and palliation to ensure quality and coherence across healthcare sectors for patients with cancer (Sundhedsstyrelsen 2022; Sundhedsstyrelsen. Forløbsprogram for rehabilitering og, 2018). The program recommends a systematic assessment of rehabilitation needs both early after diagnosis and later at regular intervals to identify individual disability challenges and needs for rehabilitation (Sundhedsstyrelsen. Forløbsprogram for rehabilitering og, 2018). However, a Danish survey concluded that only 39% of the hospital wards and outpatient clinics treating patients with cancer have adopted systematic needs assessments (Enegaaard et al. 2022). The limited adoption of needs assessments in clinical practice has also been reported in a study from the UK and Canada (Williamson et al. 2021). Moreover, the perspectives of patients and health professionals on completing needs assessment early after cancer diagnosis are only sparsely described in the literature. However, such perspectives may be valuable to better understand the poor adoption of needs assessment in clinical practice.

The aim of this study was to describe patients with non‐specific symptoms of cancer following the adoption of systematic needs assessment among those diagnosed with cancer and to evaluate the perspectives of patients, physiotherapists, and other health professionals on needs assessment.

## 2 Methods

### 2.1 Design

**FIGURE 1:**
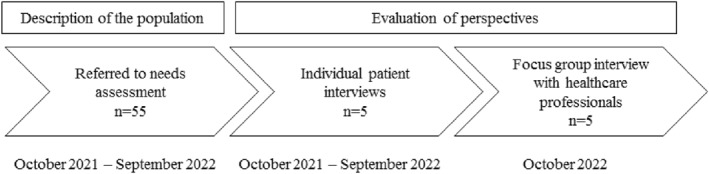
Plan of the study.

This explorative study was conducted between 1 October 2021 and 31 October 2022. We used research triangulation comprising (1) description of the population, (2) individual patient interviews and (3) focus group interview with health professionals (Figure 1).

### 2.2 Setting

Data were collected at a cancer diagnostic clinic [at Silkeborg Regional Hospital, Central Denmark Region, Denmark] serving a catchment area of approximately [237,000] residents. Each year, around 700 patients above 18 years are referred from general practice, outpatient clinics, or following hospitalisation to a diagnostic pathway for patients with non‐specific serious symptoms of cancer. Prior to referral, initial triage (diagnostic imaging and blood sampling) is performed; if no obvious cause is identified, the patient is referred to the cancer diagnostic clinic. Each patient is assigned one of the five pathway coordinators (medical secretary or nurse) who, in collaboration with one of the two internal medicine doctors, coordinates further diagnostic assessments across medical specialties. The goal is to complete all diagnostic assessments within 22 days (Sundhedsstyrelsen 2022). In Denmark, healthcare, including general rehabilitation provided by municipal healthcare centres (primary care), is tax‐financed and free of charge.

**FIGURE 2:**
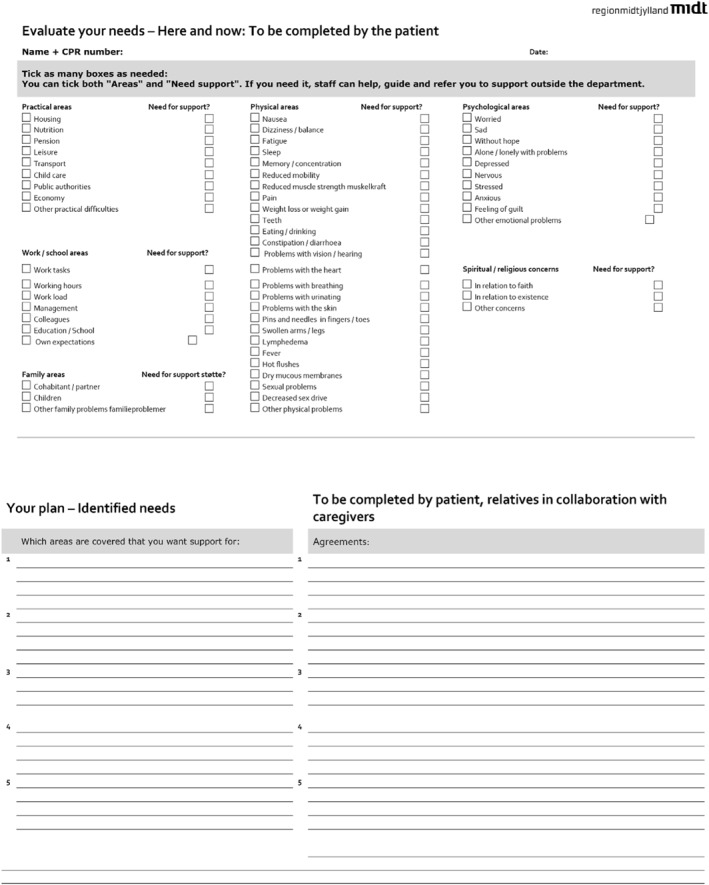
The needs assessment form (translated by the Authors from (Midtjylland 2019).

The Danish Health Authority develops standardised, interdisciplinary, cross‐sectoral, and evidence‐based disease management programmes detailing healthcare interventions, including stakeholder roles and coordination. In 2018, the Disease Management Program for Rehabilitation and Palliative Care in Cancer was updated (Sundhedsstyrelsen. Forløbsprogram for rehabilitering og, 2018). Subsequently, each of Denmark's five regions created a cooperation agreement and a needs assessment form (Sundhedsstyrelsen. Forløbsprogram for rehabilitering og, 2018). The form used in [the Central Denmark Region] aligns with WHO's widely used ICF terminology and classification, providing a holistic framework for assessing rehabilitation needs (Bickenbach et al. 2020; Midtjylland 2019) (Figure 2). The form comprises two parts: (1) six domains with 58 sub‐domains (practical, work/school, family, physical, emotional, spiritual/religious), and (2) a text field to document rehabilitation plans supporting the patient's cancer trajectory. It is a physical document owned by the patient, and must be presented during treatments, consultations, and rehabilitation (Sundhedsstyrelsen. Forløbsprogram for rehabilitering og, 2018). Although unvalidated, the form serves to identify needs, which, if present, prompt detailed assessments using validated tools, such as functional or cognitive tests (Sundhedsstyrelsen. Forløbsprogram for rehabilitering og, 2018). During the study period, we introduced systematic needs assessment into the diagnostic pathway. Patients were referred to a needs assessment by the pathway coordinator immediately after cancer diagnosis, unless excluded by the doctor due to advanced disease. Upon acceptance, a physiotherapist or occupational therapist arranged a face‐to‐face consultation within two weeks, lasting 45–60 min and conducted by a physiotherapist or occupational therapist with expertise in cancer, needs assessment, and rehabilitation. From 24 June 2022, due to reorganisation, the mode of delivery was changed from face‐to‐face to telephone and initiated directly by the physiotherapist or occupational therapist, whereas the form remained unchanged.

### 2.3 Participants

Patients diagnosed with cancer were consecutively enrolled. Patients with advanced cancer were excluded.

All patients who completed the needs assessment consented to be contacted by telephone 8–10 weeks later. A purposive sampling strategy was used to identify five patients for individual interviews who had completed a needs assessment and had rehabilitation needs (Table 1).

**TABLE 1:** Characteristics of the patients interviewed.

Patient no.	Sex	Age	Diagnosis
1	Female	71	Lymphoma
2	Male	60	Prostate cancer
3	Female	72	Lymphoma
4	Female	68	Breast cancer
5	Female	64	Breast cancer

A purposive sampling strategy was applied to identify three health professionals for a focus group interview: one doctor and two medical secretaries, who acted as primary coordinators of the needs assessments during the study period. In addition, the physiotherapist and the occupational therapist who conducted the needs assessments were included (Table 2). The first author approached the health professionals face‐to‐face and asked for consent to participate; all provided consent.

**TABLE 2:** Characteristics of the health professionals interviewed.

Health professionals	Profession	Sex	Years of experience in the cancer field
1	Occupational therapist	Female	2
2	Doctor	Female	3
3	Physiotherapist	Female	10
4	Medical secretary	Female	8
5	Medical secretary	Female	10

### 2.4 Data Collection

Descriptive data were collected from the hospital's electronic health records.

The first author conducted individual, semi‐structured telephone interviews (15–25 min) with the five patients 8–10 weeks after completion of the needs assessment. To mitigate recall bias, a trained interviewer used a semi‐structured guide with prompts to support recall of key experiences. The guide, developed by the first and second authors with a research assistant, included experience with and timing of the needs assessment and usability of the form. Interviews were documented through extensive notes.

One year after adoption of the systematic needs assessment, a research assistant (independent from the cancer diagnostic clinic) conducted a 65‐min focus group interview with health professionals at the hospital. The interview was guided by a semi‐structured interview guide developed by the first and second authors with a research assistant; it was also audio recorded, and transcribed verbatim. The main topics in the guide included perceived quality, costs per patient, feasibility and future perspectives.

### 2.5 Data Analysis

Descriptive statistics were used to present patient characteristics.

Qualitative data were analysed separately inspired by thematic analysis (Nowell et al. 2017): (1) The first author familiarised with data by reading notes and transcripts, (2) the first and third authors developed and discussed initial codes and themes and (3) the first, second and third authors reviewed and defined themes, and data from patient interviews and focus group interview were triangulated. If consensus on the themes was not achieved, further discussions were conducted.

### 2.6 Ethics Approval and Patient Consent Statements

This study complied with principles of the World Medical Association Declaration of Helsinki Ethical Principles for Medical Research involving Human Subjects. According to legislation and the Act on Biomedical Research Ethics Committee System, qualitative research does not require approval from an ethics committee (§14 Section 2) (Sundhedsministeriet 2020). All informants provided written informed consent to participate in the interviews. In accordance with the guidelines of the Data Protection Agency, the hospital management granted permission for data collection from electronic health records.

## 3 Results

**FIGURE 3:**
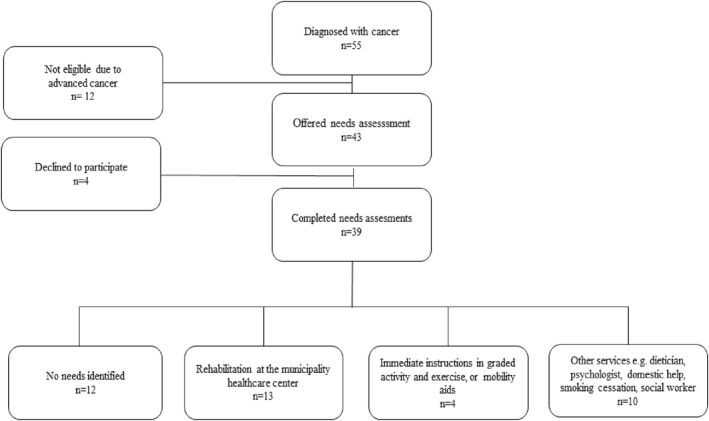
Flow of patients diagnosed with cancer during the study period.

During the study period, 633 patients were referred to the cancer diagnostic clinic. Of these, 55 were diagnosed with cancer, and 43 were offered a needs assessment. Among those who completed the needs assessment (*n* = 39), 27 reported various rehabilitation needs, whereas 12 reported no needs, none of them with signs of cognitive or emotional impairment (Figure 3). The characteristics of the 55 patients diagnosed with cancer are presented in Table 3.

**TABLE 3:** Characteristics of the 55 patients diagnosed with cancer.

	Not eligible *n* = 12	Declined *n* = 4	Completed *n* = 39
Male sex, *n* (%)	9 (75)	1 (25)	22 (56.4)
Age, mean (min‐max)	74 (56–87)	78 (75–79)	67 (33–85)
Cancer type, *n* (%)			
Prostate	1 (8.3)	0 (0)	5 (12.8)
Breast	2 (16.7)	0 (0)	6 (15.4)
Malignant melanoma	1 (8.3)	0 (0)	1 (2.6)
Lymph node	2 (16.7)	3 (75)	6 (15.4)
Gastrointestinal	4 (33.3)	1 (25)	5 (12.8)
Endocrinological	0 (0)	0 (0)	11 (28.2)
Other	2 (16.7)	0 (0)	5 (12.8)
Mode of delivery, *n* (%)			
Face‐to‐face^a^			11 (28.2)
Telephone^b^			28 (71.8)

^a^ October 1. 2021—June 24. 2022.

^b^ June 25—September 30. 2022.

Three themes emerging from the data analysis are described below.

Theme 1 Early needs assessment and rehabilitation when diagnosed with cancer is meaningful to patients and health professionals.

Overall, patients and health professionals were satisfied with the needs assessment early after diagnosis. Patients were comfortable with the needs assessment early after diagnosis as it provided coherence in their cross‐sectorial cancer trajectory. The early timing provided an important sense of support during a period of uncertainty and emotional strain following the unexpected news of disease. One patient explained as follows:

The timing was very appropriate. It was really fine for me. I can only recommend it. My illness came out of the blue, so having my needs addressed from the start made me happy.

(Patient participant 1)

Although patients reported different needs, they found the needs assessment relevant, particularly for obtaining information on municipal rehabilitation support. It also reassured them that their needs would be addressed. Patients valued the emphasis on ‘activity and participation’, ‘environmental factors’, and ‘personal factors’, rather than exclusively on ‘body functions and structures’.

Patients perceived that the dialogue focused on their options, even shortly after a cancer diagnosis, a time when they often felt a lack of control:

It was a good experience. Time was set aside to reflect on things in my daily life. It was a very nice conversation. When you see the doctor, there is a special focus, for example on chemotherapy. It was okay to be lifted away from the heavy and serious topics.

(Patient participant 2)

For some patients, the needs assessment was described as particularly helpful because it provided clear guidance, emotional reassurance, and concrete information on available rehabilitation options. These aspects were perceived as directly contributing to their rehabilitation process by creating a sense of structure and continuity in care. One patient expressed as follows:

I felt I was taken by the hand to move on.

(Patient participant 5)

The health professionals experienced the needs assessment and rehabilitation plan early after diagnosis as highly meaningful for patients and as well as helpful in the workflow in the cancer diagnostic clinic. There was interdisciplinary consensus that it was appropriate to perform needs assessments and that it had no negative consequences from a healthcare perspective. The health professional's satisfaction was fundamentally influenced by a multitude of positive patient experiences, and the desire to make a difference as a health professional. In addition, the societal consequences of the needs assessment were considered particularly important, such as patients' connection to the labour market. As described by a doctor:

So, I think as a doctor that many times, I have had the feeling that there might well be a need for assistive devices or training, but we have a time limit and have to achieve a whole lot of things in relation to diagnosis, so we have to sort out something. The provision of physiotherapy and occupational therapy to the patient is welcome. We address something that the patients benefit from which other professionals do not have the opportunity to honor.

(Participant 2, Doctor)

In addition, one of the medical secretaries expressed:

… by introducing the needs assessment, patients benefit from something which other professionals do not address. If I was a patient, I would have all the offers available for me to handle the diagnosis and treatment, and so on. Rehabilitation needs and plans make perfect sense.

(Participant 5, Medical secretary)

Theme 2 Organisational barriers to offering rehabilitation.

The patients and health professionals also addressed barriers and suggestions for optimising efforts in future clinical practice. Three of the five patients expressed a need for a more individual rehabilitation plan rather than group‐based rehabilitation, which is common in many municipal healthcare centres. One patient expressed it this way:

I had hoped to be able to take part in rehabilitation, which was more suited for me. I wish there were better options. Maybe a little more individualized.

(Patient participant 1)

Another patient asked for rehabilitation opportunities with a broader focus than physical activity and exercise as it was challenging both to be seriously ill and severely exhausted.

Time and resources constituted a barrier for doctors and pathway coordinators in completing needs assessments early in the patient's pathway. Consequently, they valued the contributions of physiotherapists and occupational therapists in carrying out these assessments.

During the study period, a reorganisation meant that patients were contacted directly by a physiotherapist or an occupational therapist instead of the pathway. This reorganisation turned out to have an unexpectedly positive effect on the completion of needs assessments. Health professionals therefore agreed that in future clinical practice, physiotherapists and occupational therapists should contact patients directly to plan the needs assessment. The health professionals believed that this would lead to better coherence in the patient pathways and thereby ultimately benefit the patients. The health professionals were also concerned about patients with diagnoses other than cancer because many of these patients had evident but unmet rehabilitation needs:

I think it could be very good if the service was made available to other patient groups as well. I mean to those who are not diagnosed with cancer.

(Participant 2, Doctor)

The health professionals agreed that clinical staff satisfaction with the needs assessment could be improved if the offer was extended to a large number of patients who do not have cancer but still have rehabilitation needs.

Theme 3 The needs assessment form is useful to provide information and guide conversations.

In general, patients found that the form covered their rehabilitation needs.

They perceived the assessment as comprehensive, acknowledging that while some topics were not relevant to them, they could be important for other patients:

I think the needs assessment form covered a lot. It wasn’t necessary to talk about my network, but it may be relevant to other patients

(Patient participant 5)

Three of the patients completed the physical form on their own before the consultation with the therapist, and two patients completed it together with the therapist. The physiotherapists and occupational therapists expressed that the form was difficult for patients to fill out on their own but experienced it to be a relevant tool to guide conversations:

I completely understand if patients react: ‘Oh my God, how do I complete this needs assessment form?’ It's not that intuitive, is it? But using the needs assessment form as a tool to guide the conversation ensures that all needs are expressed by the patient.

(Participant 3, Physiotherapist)

One patient actively used the physical form, as was its intention, during her cancer treatment:

I was given a needs assessment form. I still have it at home. That’s nice. When you're in the situation I'm in, you quickly forget something. I use the form as a reminder and keep it in my car, so I have it ready when visiting different hospitals.

(Patient participant 1)

Health professionals considered a cross‐departmental use highly relevant because it could inform general practitioners and/or health professionals in the municipal healthcare centres about the patient's biopsychosocial situation and everyday life.

## 4 Discussion

This exploratory study, which to our knowledge is the first in this field, characterised a group of 55 patients with cancer and identified their rehabilitation needs. Further, the study showed that early systematic needs assessment is considered valuable and appreciated by patients and healthcare professionals. Attention should be given to physical, psychological, spiritual/religious and social needs, aligning with WHO's definitions of rehabilitation and palliation (Sundhedsstyrelsen. Forløbsprogram for rehabilitering og, 2018; WHO 2023, 2024). These results demonstrate how adoption of early needs assessments can guide effective, daily life‐focused rehabilitation for cancer patients.

In our study, the doctor assessed that 12 patients (22%) were not eligible to complete a needs assessment due to advanced cancer. A systematic review documented that patients with advanced cancer had a wide range of unmet care needs (both physical and psychological) (Wang et al. 2018), and that these unmet needs should be comprehensively assessed (Wang et al. 2018). Since needs assessment was new in the cancer diagnostic clinic, it may have influenced the doctor's assessment of who was eligible to receive the needs assessment. An identical low number of patients with advanced cancer referred to rehabilitation has been documented previously in the literature identifying that oncologists consider prognosis as a significant barrier to rehabilitation referral (Spill et al. 2012). Therefore, this will be a focus area in our future clinical practice, and the physiotherapists and the occupational therapists with their specific knowledge within disability and rehabilitation can play an important role in this discussion.

In the present study, needs assessment was offered to 43 out of 55 patients (78%), and of them 39 out of 43 (91%) completed it. This number is higher than reported in a national Danish survey where only half of the patients were offered a needs assessment (Enegaaard et al. 2022). A Danish survey of 32 inpatient wards and outpatient clinics found that 75% estimated that at least half of patients were offered a systematic needs assessment, based on nurses' estimates rather than exact data. This lower percentage than the precise data from our cancer diagnostic clinic, obtained from electronic health records, may reflect differences in setting, as needs assessments are generally easier to integrate during planned outpatient consultations than acute hospital admissions. In total, 27 patients in our study stated their needs and were referred to a variety of services. This testifies to the importance of assessing functioning from a holistic perspective. It also emphasises the importance of health professionals being able to refer patients to the right support and services. This is supported by a survey conducted among cancer nurses in the UK and Canada, which revealed that one of the barriers to completing needs assessments’ was the inability to refer patients to appropriate support and services (Williamson et al. 2021).

Reorganisations during the study shifted the needs assessment from face‐to‐face to telephone consultations and with physiotherapists and occupational therapists engaging patients directly rather than via a pathway coordinator. This increased the number of needs assessments from 11 in nine months to 28 in three months. This was an unexpected positive result, supporting a previous study documenting that telephone consultations can be implemented to a high degree with good patient acceptance in patients with cancer completing a needs assessment (Askjær et al. 2023). Further, it has been described that it can be easier and more comfortable for both patients and health professionals to engage in discussions about sensitive and complex topics, such as psychosocial difficulties, over the telephone (Askjær et al. 2023). This method can create a more relaxed atmosphere that allows for open and honest conversations that can otherwise be challenging in face‐to‐face interactions (Williamson et al. 2021). However, we do not know whether the telephone consultation or the direct interaction between the therapist and the patient triggered the increase in the number of completed needs assessments.

Overall, the interviews revealed that needs assessment was generally well‐received by both patients and health professionals. This partially contrasts with the aforementioned survey, finding lack of confidence, privacy, and resources, but especially lack of time and staff shortages as the biggest barriers to adopting needs assessments (Williamson et al. 2021). The latter is in line with our study, where the health professionals appreciated the integration of therapists in the cancer diagnostic clinic as they facilitated the completion of needs assessments that were previously unfeasible due to limitations in time and staff resources.

Further, we found that both patients and health professionals underlined the importance of and appreciated that the needs assessment embraced a level of functioning in everyday life in addition to “body functions and structures”. Both patients and health professionals found the form understandable. Patients were satisfied with the timing of the needs assessment early after diagnosis, and they appreciated the focus on needs related to all five ICF components. This is in line with the international endorsement of ICF as a valuable way to describe and measure health and disability (WHO 2013). The health professionals indicated that the form was used more as a conversation guide rather than a checklist. Surprisingly, only one of the interviewed patients actively used the form as a physical document during the cancer trajectory. The responsibility for the use of this form during the cancer trajectory lies within the health professionals (Sundhedsstyrelsen. Forløbsprogram for rehabilitering og, 2018). To support coherent use across the cross‐sectoral cancer trajectory, the form should be promoted among healthcare professionals through targeted training, workflow integration, regular reminders, and demonstrations of its value for rehabilitation and patient care.

The present study has some limitations. Firstly, given the limited sample of 39 patients who completed a needs assessment, we refrained from comparing cancer types or exploring differences in identified needs. We acknowledge that the impact of cancer varies by type and prognosis, and that individual needs are further shaped by factors such as age, comorbidities, lifestyle, social context, education, and cultural background (Sundhedsstyrelsen. Forløbsprogram for rehabilitering og, 2018). Secondly, patient interviews were conducted 8–10 weeks after the needs assessment to allow patients to initiate rehabilitation in municipal healthcare centres. Even though recall bias cannot be entirely excluded, patients were able to reflect on the assessment and its long‐term relevance. Thirdly, only five patient interviews were conducted, and thus, data saturation cannot be assured. However, the patient interviews provided valuable insights to argue for introducing needs assessments early in a cancer trajectory. Finally, the perspectives of patients with no identified needs (*n* = 12) could have provided interesting information to our results.

We believe, however, that our results can inspire other cancer diagnostic set‐ups, including organ‐specific cancer pathways. Symptoms and signs of cancer and related disability often evolve over time and needs assessments and rehabilitation plans are thus important regardless of the diagnostic set‐up. Before the study period, needs assessment was not a part of the usual practice in our cancer diagnostic clinic. This is in line with the current literature reporting that needs assessments and rehabilitation plans are often not provided or coordinated, for example, during diagnostics, but are introduced and offered later in the patient's trajectory (Wade 2016), despite national recommendations (Sundhedsstyrelsen. Forløbsprogram for rehabilitering og, 2018) and well‐known positive outcomes of early onset rehabilitation (Nottelmann et al. 2021). This may be explained by the fact that understanding the impact of disability often plays only a minor role in hospital‐based healthcare (Wade 2016). This is challenging since disability affects most patients living with or surviving cancer (Holm et al. 2012; Neo et al. 2017; Thorsen et al. 2011). In our cancer diagnostic clinic, the needs assessment has now been adopted into clinical practice and is provided by a physiotherapist or an occupational therapist to all patients early after cancer diagnosis. Thus, the results of this study have optimised our clinical practice by completing systematic needs assessments, and we believe that this is a step in the right direction compared to previous practice described both nationally and internationally (Enegaaard et al. 2022; Williamson et al. 2021).

This explorative study provides an overview of how a population of 55 patients diagnosed with cancer is characterised and what rehabilitation needs they have. Further, the study documents that early systematic needs assessments are valuable and well‐received by both patients and healthcare professionals. Attention should be paid to the patient's needs for interventions in relation to physical, psychological, spiritual/religious and social issues, in accordance with the WHO definition of rehabilitation and palliation (Sundhedsstyrelsen. Forløbsprogram for rehabilitering og, 2018; WHO 2023, 2024).

## 5 Implications for Physiotherapy Practice

These results are useful in clinical practice as they show one way to implement early systematic needs assessment and highlight the importance of early rehabilitation focused on daily life in patients diagnosed with cancer. Physiotherapists play an important role in the multidisciplinary team in cancer diagnostic pathways as they have specific knowledge about everyday life and rehabilitation seen through a holistic lens.

During the study, the physiotherapists and the rest of the multidisciplinary team in the cancer diagnostic clinic became aware of patients diagnosed with another serious disease or without a confirmed diagnosis, many of whom appeared to experience disability. At that time, needs assessments were only offered to patients diagnosed with cancer, in line with a former recommendation (Sundhedsstyrelsen. Forløbsprogram for rehabilitering og, 2018). Since then, a new recommendation has highlighted that all patients referred to a cancer pathway, regardless of diagnosis, are offered a needs assessment to plan their further trajectory based on their health condition, skills, resources, self‐care ability and motivation (Sundhedsstyrelsen 2022). We have recently documented that functioning difficulties are present in patients referred to our cancer diagnostic clinic, regardless of whether they receive a cancer diagnosis. This finding supports the need for early needs assessment prior to diagnosis (Bloch‐Nielsen et al. 2025). However, we recommend that the current form should be tested, evaluated and, if necessary, adapted before being applied to patients with different cancer types and stages or without a confirmed diagnosis across various settings. For the physiotherapy profession, understanding the relationship between needs assessment dimensions and patient outcomes such as recovery, quality of life and satisfaction is essential for developing more targeted, patient‐centred rehabilitation strategies and improving care effectiveness.

## Author Contributions

All authors have made a substantial contribution to the conception and design of this study. Observations were conducted by Helene Nørgaard Kristensen and Jannie Rhod Bloch‐Nielsen, and individual participant interviews were conducted by Helene Nørgaard Kristensen. Helene Nørgaard Kristensen, Rikke Aarhus and Jannie Rhod Bloch‐Nielsen conducted the data analysis in collaboration. An initial manuscript draft was prepared by Helene Nørgaard Kristensen, Thomas Maribo and Anne Mette Schmidt. All authors revised the manuscript critically for important intellectual content, approved the version to be published and agreed to be accountable for all aspects of the work in ensuring that questions related to the accuracy or integrity of any part of the work are appropriately investigated and resolved.

## Funding

The authors have nothing to report.

## Conflicts of Interest

The authors declare no conflicts of interest.

## Data Availability

The data that support the findings of this study are available from the corresponding author upon reasonable request.
